# Simulation-guided tunable DNA probe design for mismatch tolerant hybridization

**DOI:** 10.1371/journal.pone.0305002

**Published:** 2024-08-22

**Authors:** Pallavi Bugga, Vishwaratn Asthana, Rebekah Drezek

**Affiliations:** Department of Bioengineering, Rice University, Houston, Texas, United States of America; Rutgers Biomedical and Health Sciences, UNITED STATES

## Abstract

The ability to both sensitively and specifically assess the sequence composition of a nucleic acid strand is an ever-growing field. Designing a detection scheme that can perform this function when the sequence of the target being detected deviates significantly from the canonical sequence however is difficult in part because probe/primer design is based on established Watson-Crick base-pairing rules. We present here a robust and tunable toehold-based exchange probe that can detect a sequence with a variable number of SNPs of unknown identity by inserting a series of controlled, sequential mismatches into the protector seal of the toehold probe, in an effort to make the protector seal “sloppy”. We show that the mismatch-tolerant system follows predicted behavior closely even with targets containing up to four mismatches that thermodynamically deviate from the canonical sequence by up to 15 kcal/mole. The system also performs faithfully regardless of the global mismatch position on either the protector seal or target. Lastly, we demonstrate the generalizability of the approach by testing the increasingly mismatch-tolerant protectors on HIV clinical samples to show that the system is capable of resolving multiple, iteratively mutated sequences derived from numerous HIV sub-populations with remarkable precision.

## Introduction

The detection of sequence-specific nucleic acids is a growing field not only in molecular biology but also the clinic [1,2]. Increasing demand has been placed on nucleic acid-based detections probes to not only be sensitive and specific, but also robust and tunable. However, designing a probe *in silico* that meets these criteria when the sequence of the target being detected deviates significantly from the canonical sequence is incredibly difficult, in part because probe design is based on established Watson-Crick base-pairing rules [3]. Tools like Next Generation Sequencing (NGS) can be used to determine the exact sequence identity of the region of interest, but such methods are often time-intensive and costly.

While many clinically relevant mutations and single-nucleotide polymorphisms (SNPs) are well known–allowing for the design of specific detection probes–the identity of many SNPs are unknown *a priori*, thereby preventing the design of appropriate diagnostic probes [4]. Further, more than one SNP may be present in a region of interest, making reliable detection all the more difficult. This is especially true for hypermutable sequences, including viral genomes, VDJ recombination regions, and trinucleotide repeats [5–9]. There are numerous applications that require controlled targeting of an unknown or hypermutable sequence that deviates from a canonical sequence. These applications include, for example, the use of mismatch-tolerant hairpin probes to broadly bind across a mutagenic genome to identify an unknown species, use of mismatch-tolerant primers to bind to and amplify a single hypermutable target, direct probe binding and SNP detection, and site-directed insertion/mutagenesis of multiple SNPs [10–14]. As an illustrative example, if one were to attempt to design a probe that selectively binds to a region known to harbor gain- or loss-of-function SNPs, without knowing the number or identity of the SNPs *a priori*, design options would be limited given that the majority of detection schemes center around the probe binding to a known SNP against a wildtype. The tunable probes discussed here are also advantageous for simultaneously interrogating targets that are dissimilar, but that still retain a high degree of sequence homology or conservation (such as the 16s rRNA gene of the bacterial genome), as they preclude the need to design probes specific to every sequence variant.

We present a toehold-based probe system that can be intelligently designed *in silico* to detect a sequence with a variable number of SNPs of unknown identity, or a group of sequences with a variable degree of sequence similarity, using thermodynamic cutoffs. Toehold probes are incredibly sensitive and specific nucleic acid-based detection tools that operate reliably under a wide range of salts and temperatures. In addition, relatively simple *in silico* design tools can be used to ensure toehold probes detect SNPs with high selectivity versus wildtype. However, because current iterations of the probe-protector system are designed to be ultra-specific, the toehold probes can only tolerate a known SNP either in the protector or target strand [3,15]. When more than one SNP is present or the sequence is unknown, the system begins to deviate from its expected behavior. We show that introducing a series of controlled mismatches into the protector of the toehold probe allows the protector to tolerate target sequences with an increasing number of mismatches/SNPs. By extension, the identities of these SNPs do not need to be known *a priori* to ensure robust detection.

While most nucleic acid-based detection probes are designed based on sequence homology to ensure specific binding, the mismatch-tolerant protectors described herein rely predominantly on Gibbs free energy (ΔG°) cutoffs. Specifically, a target sequence with more mismatches than the tolerant protector will have a standard free energy of hybridization to the probe that is less favorable than the protector-probe duplex. As a result, the mismatched target cannot displace the protector and bind the probe. Conversely, a target with less mismatches and a more favorable ΔG° than the tolerant protector *can* displace the protector and bind to the probe. Despite using ΔG° cutoffs, the mismatch-tolerant probe-protector system is still resilient to GC content, due to the requirement of target/protector sequence homology to probe. Of note, DNA hairpin probes–which also confer some mismatch tolerance–are generally less stable and consistent than the protected probe scheme listed here, often leading to increased nonspecific activation and off-target effects [15].

**Fig 1:**
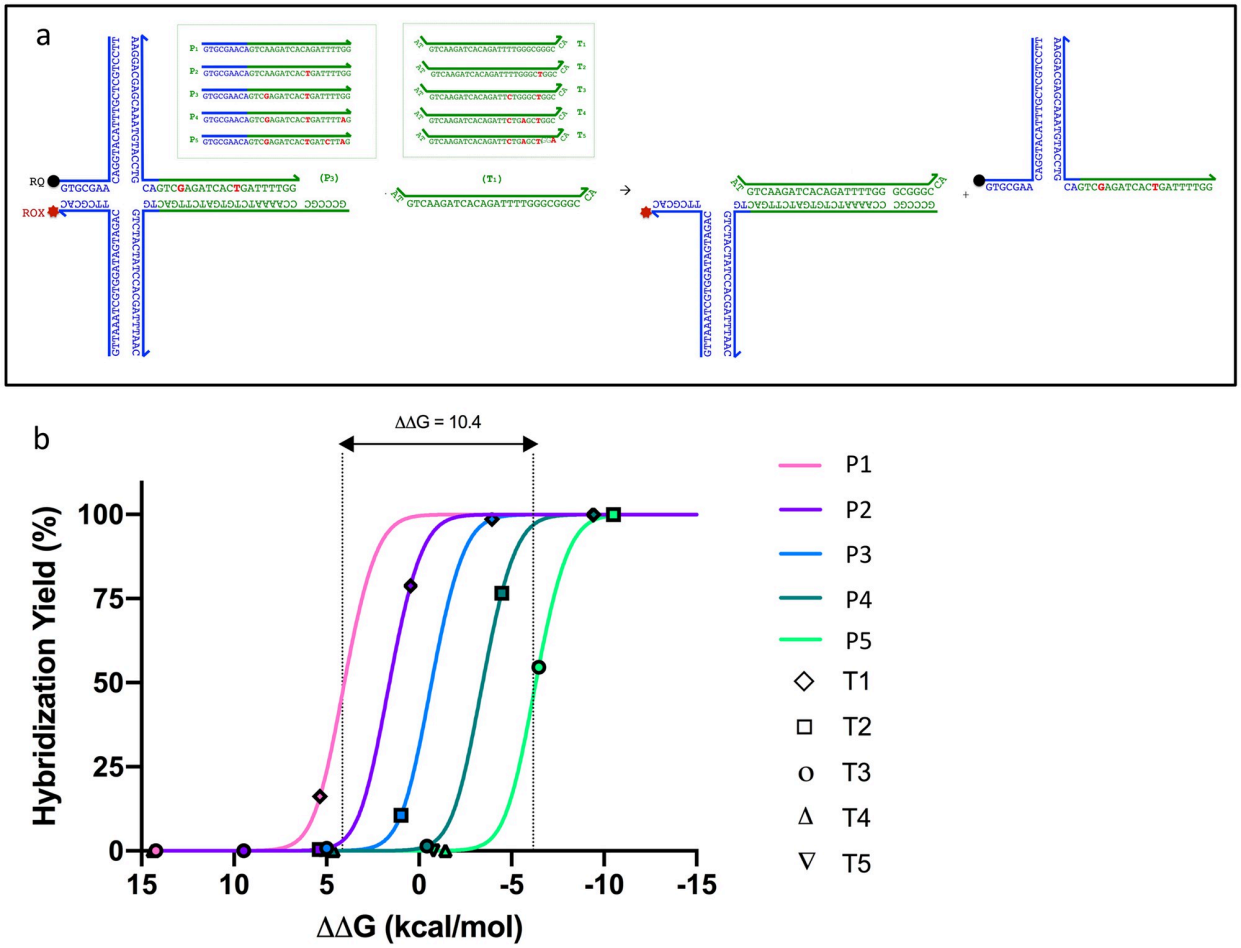
Schematic of mismatch-tolerant protector design using X-probes. **A)** Schematic of an X-Probe displacement reaction in the presence of a more complementary target, with Protector 3 (P_3_) and Target 1 (T_1_) used as examples. The X-probes contain modifiable protector and probe regions (depicted in green), as well as universal fluorophore (ROX, 588–608 nm)- and quencher (RQ)-functionalized regions (depicted in blue). The thermodynamically relevant portions of the sequences for the set of 5 mismatch protectors (P_1-5_) and 5 targets (T_1-5_) are shown–these include the “horizontal” regions of the X-probe immediately adjacent to the fluorophore/quencher label (as these strands function as the probe-protector toehold) but not the “vertical portions” of the X-probe, as these latter regions are not considered part of the three-strand approximation when determining hybridization efficacy. **B)** Theoretical hybridization yields for five increasingly tolerant protectors (P_1-5_) against five targets (T_1-5_). X-axis corresponds to the free energy difference between the probe-protector and probe-target, centered around the free energy difference between the probe and P_3_, the latter of which has been set to zero for illustrative purposes. A target with more mismatches against the probe (relative to the protector) has a positive ∆∆G and will be less likely to displace the protector. Conversely, a target with less mismatches and a more favorable ΔG° than the mismatch-tolerant protector can displace the protector and bind to the probe (ex. T_3_ to P_4-5_).

Given that the average standard free energy penalty of a single-mismatch ranges from approximately 1.5–6.5 kcal/mole, and further that the hybridization efficiency of probe-protector displacement is sigmoidal in nature with respect to the standard free energy, with exponential-like qualities in the region between +5 to -5 kcal/mole (for or system), we can expect sharp cutoffs in hybridization yield for a set probe-protector complex with each target mismatch iteration [15,16]. Accordingly, by using a set of increasingly mismatch-tolerant protectors that span a sufficiently wide ΔG° range, the approximate ΔG° of the probe-target complex can be approximated and the identity of the target and its corresponding SNPs inferred. A schematic of the design, using a set of 5 increasingly mismatch-tolerant protectors (P_1-5_) and 5 targets with variable number of SNPs (T_1-5_), is displayed in Fig 1.

## Results

### Mismatch-tolerant protector performance

Five target [T_1-5_] and five protector sequences [P_1-5_] were designed, each with an increasing number of SNPs relative to the consensus probe (T_1_ = zero mismatches → T_5_ = four mismatches; P_1_ = zero mismatches → P_5_ = four mismatches) (**S1a and S1b Table in** S1 File). These SNPs were inserted at spaced intervals on both the protector and target. Further, where possible, the targets and protectors were designed with the following thermodynamic constraints in mind,

$$\Delta \text{G}{}^{\circ}{\text{of}\;\text{Probe-P}}_{\text{x}}\leq \Delta \text{G}{}^{\circ}{\text{of}\;\text{Probe-T}}_{\text{x}}\leq \Delta \text{G}{}^{\circ}{\text{of}\;\text{Probe-P}}_{\text{x}+1},$$ (1)

wherein the ΔG° values were determined via NUPACK. Given the sigmoidal nature of toehold displacement, where a marginal variation in ΔΔG° (defined as the ΔG° gap between [Probe-T] and [Probe-P]) significantly alters hybridization yields around null, we can expect sharp cutoffs in displacement within each ΔG° interval delineated by equation [1]. This is especially true when the typical ΔG° penalty of a single mismatch varies anywhere between 1.5–6.5 kcal/mole, enabling the compartmentalization of individual mismatches within the intervals specified by each increasingly mismatch-tolerant protector [15,16].

To facilitate testing and reduce potential costs of using multiple mismatch-tolerant protectors in parallel, X-probes were utilized [17]. X-probes are conditionally fluorescent nucleic acid probes in which the two functionalized universal detection oligonucleotides (fluorescent [ROX] and quencher [RQ]) are decoupled from the probe-protector-target complex. Consequently, the same ROX and RQ fluorophore and quencher species can be used with any combination of probe-protector detection pairs. In addition, the reaction mechanism of the X-Probe is similar to that of the toehold probe and well characterized [17].

**Fig 2:**
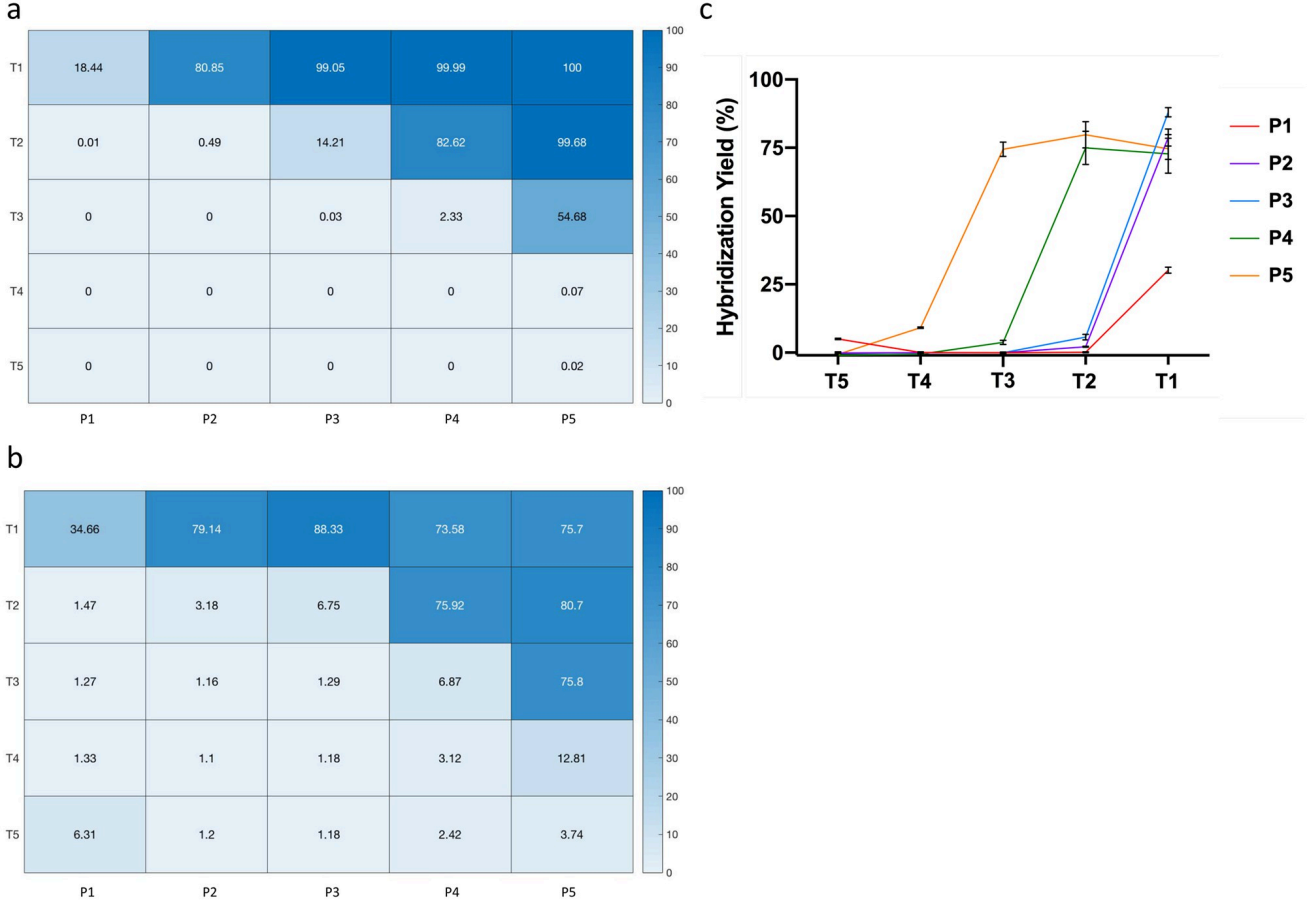
Experimental versus theoretical yields of increasingly mismatch-tolerant hybridization probes. **A)** Expected/theoretical yields for five target (T_1-5_) and five protector sequences (P_1-5_), each with an increasing number of SNPs relative to the consensus probe (T_1_ = zero mismatches → T_5_ = four mismatches; P_1_ = zero mismatches → P_5_ = four mismatches). Values are given in terms of percent yields. **B)** Experimental yields for five target (T_1-5_) and five protector sequences (P_1-5_), each with an increasing number of SNPs relative to the consensus probe (T_1_ = zero mismatches → T_5_ = four mismatches; P_1_ = zero mismatches → P_5_ = four mismatches). Values are given in terms of percent yields. **C)** A linear plot of the data depicted in [B], demonstrates that hybridization of increasingly mismatched targets relative to progressively mismatch-tolerant protectors follows strict ΔG° thresholds. Error bars represent the standard deviation of triplicate conditions.

Results of experiments testing T_1-5_ and P_1-5_ in Fig 2 confirm that toehold displacement of increasingly mismatched protector strands can be accurately predicted using current simulation methods. The expected/theoretical yields of the various protector-target complexes is depicted in Fig 2a, and appears to correlate strongly with the experimental yields depicted in Fig 2b (adjusted-r^2^ = 0.94, RMSE = 9.62). Experimental yields were calculated by normalizing each displacement against a positive control (the fluorescent strand only, or F_max_) with

$$Yield\left(\%\right)=\frac{x-F_{B}}{F_{max}-F_{B}}\times 100$$

and where *x* corresponds to the end-point fluorescence of the reaction, and *F*_*B*_ corresponds to the fluorescent background (the quenched X-probe without target added). Additionally, Fig 2c, a linear plot of Fig 2b, demonstrates that hybridization and corresponding signal detection of increasingly mismatched targets relative to progressively mismatch-tolerant protectors follows strict cutoffs that correspond to ΔG° thresholds.

It should be noted that protectors P_2_ and P_3_ behave nearly identically in terms of their displacements to targets T_1-5_. For initial experiments, attempts were made to insert mismatches to not only follow the thermodynamic constraints depicted in equation [1] but also to be somewhat evenly spaced from one and another to minimize unintended deviations from predicted displacement. Because these design considerations are not mutually exclusive, the ΔG° gap between protectors P_2_ and P_3_ inadvertently ended up being smaller than the ΔG° gap between targets T_1_ and T_2_ leading to a slight deviation from equation [1], with the ΔG of Probe-T_1_ < Probe-P_2_ < Probe-P_3_ < Probe-T_2_ and a subsequent stagger (**S1 Table in** S1 File). As a result, the thermodynamic cutoffs of protectors P_2_ and P_3_ overlap. The effects of this overlap however were accurately predicted via simulation.

Finally, kinetic traces of mismatch-tolerant protector displacement indicate that the kinetics of the reaction are in agreement with previously characterized toehold probe reaction rate constants, and that increasing the number of mismatches in the target or protector strands has no appreciable effect on the rate of the strand displacement reaction, even when such mismatches occur on the target toehold, as is the case with targets T4 and T5 (S1a–S1e Fig) [15]. A table of all probe, protector, and target sequences and their respective ΔG° of hybridization can be found in **S1 and S2 Tables in** S1 File.

### Impact of global mismatch position

**Fig 3:**
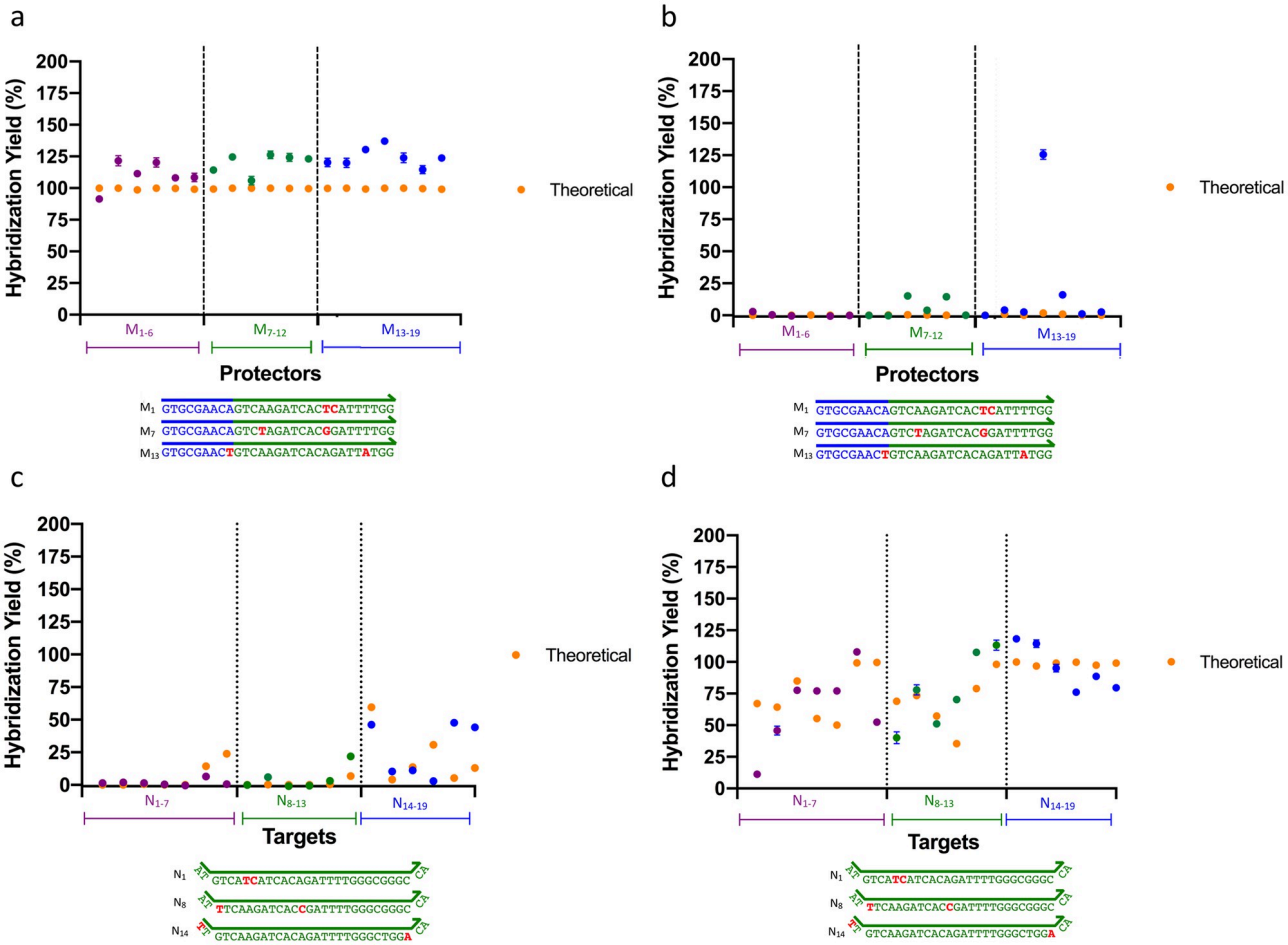
Assessing the impact of global mismatch position on mismatch-tolerant protector performance. P_3_ protectors with two mismatches placed either immediately adjacent to one and another, corresponding to zero bp of separation (M_1-6_), seven bp apart (M_7-12_), or greater than 16 bp apart (M_13-19_) were tested against a **A)** T_1_ and **B)** T_3_ target. The representative protector sequences provided for M_1_, M_7_, and M_13_, include only the thermodynamically relevant portions of the protector–that is, the “horizontal” region of the X-probe immediately adjacent to the fluorophore/quencher label (as this portion functions as part of the probe-protector toehold). The sequences do not incorporate the “vertical portions” of the X-probe, as these regions are not considered part of the three-strand approximation when determining hybridization efficacy Similarly, T_3_ targets with two mismatches spaced as described above–that is, zero bp apart (N_1-6_), seven bp apart (N_7-12_), or greater than 16 bp apart (N_13-19_)–were tested against a **C)** P_2_ and **D)** P_4_ protector. Experimental yields versus simulation demonstrate the robustness of the mismatch tolerant hybridization system to global mismatch position, save for a few outliers. Error bars represent the standard deviation of triplicate conditions.

Because the requirement to space mismatches evenly on the protector can interfere with the design of consistently spaced ΔG° gaps, and the position of multiple mismatches is not guaranteed to be evenly spaced on the target, we next assessed whether the global position of a mismatch, either on the protector or target, would disrupt predicted hybridization patterns and, in turn, the selective displacement expected within each ΔG° interval. Accordingly, mismatches were inserted at set locations on the target and protector strand. Specifically, P_3_ protectors with two mismatches placed either immediately adjacent to one and another (M_1-6_), corresponding to zero bp of separation, seven bp apart (M_7-12_), or greater than 16 bp apart (M_13-19_) were tested against a T_1_ and T_3_ target (Fig 3a and 3b, **S3 Table in** S1 File). Similarly, T_3_ targets with two mismatches spaced as described above–either zero bp apart (N_1-7_), seven bp apart (N_8-13_), or greater than 16 bp apart (N_14-19_)–were tested against a P_2_ and P_4_ protector (Fig 3c and 3d, **S4 Table in** S1 File).

Fig 3 demonstrates that the presence of two mismatches stacked together, spaced 7 base pairs apart, or at the periphery of the protector strand (>16 base pairs apart) does not significantly alter select displacement of the P_3_ protector (P_3_ = two mismatches) relative to the T_1_ and T_3_ target (Fig 3a and 3b), and similarly does not significantly alter select displacement of the T_3_ target (T_3_ = two mismatches) relative to the P_2_ and P_4_ protector relative to simulation (Fig 3c and 3d). The bounding strands tested against the P_3_ protector and T_3_ target were chosen because their upper and lower thermodynamic affinities constrain the ΔG° range of the various mismatches.

Overall, experimental versus simulation data confirms that the position of the mismatches on either the target or protector does not appreciably affect predicted base-pairing thermodynamics and corresponding select displacement outside the specified ΔG° intervals, save for one outlier (M_16_ + T_3_). With the inclusion of said outlier, the adjusted r^2^ and RMSE equal 0.83 and 18.58, respectively. Upon elimination of the outlier, the adjusted r^2^ and RMSE become 0.89 and 15.00, respectively. Experimental yields for this experiment were calculated using the same method described above for Fig 1.

Overall, the representative 101 combinations of mismatched protectors and targets follow predicted behavior closely (r^2^ = 0.91, RMSE = 13.46 without the above delineated outliers), validating the use of a set of increasingly mismatch-tolerant protectors that span a sufficiently wide ΔG° range to approximate the ΔG° of a probe-target complex. The set of protectors devised for the aforementioned experiments alone span a ΔG° range of 15 kcal/mol and together can accurately resolve variously mismatched targets within this interval with high fidelity.

### Tests on hypermutable clinical correlates

Having validated the system against samples containing a single dominant sequence with variable numbers and identities of SNP, we next sought to test how the mismatch tolerant hybridization system would behave in the presence of multiple sub-populations, each harboring sequences containing a unique SNP signature. The former scenario is typically encountered when SNPs are well conserved—such as in eukaryotes—in which the distribution of the unique SNP-containing sequence is generally expected to be present either at 100% (homozygous) or 50% (heterozygous) depending on the nature of that particular mutation [18]. The latter situation, however, can be found in certain species with hypermutable sequences, such as viruses, in which several sequences with an iterative number of acquired SNPs of unknown identity can be typically expected to co-exist; these mutated genomes are often referred to as quasi-species, and have been investigated in the context of viral resistance to therapy [6]. In the case of HIV for example, patients who have developed resistant strains as a result of chronic, unchecked infection or treatment noncompliance, will either harbor a primary HIV genome that deviates from the endemic sequence or carry multiple HIV subpopulations as observed in this study–information that can help determine prognosis and guide therapy [19].

To validate the robustness of the mismatch-tolerant protectors in such a setting, we tested the system on HIV clinical sequences known to be highly mutable and generally difficult to characterize. HIV RNA was extracted from serum samples collected from HIV-infected patients and reverse transcribed into cDNA. Known hypermutable segments were sequenced using Next Generation Sequencing and a single region with significant heterogeneity in both number and type of SNPs was selected for testing using the mismatch-tolerant protector system (**S5 Table in** S1 File). Based on sequencing data of the *pol* gene, taken from one of three patient samples, three unique and related HIV subtypes or subpopulations were observed (Targets 1–3)–at a normalized prevalence of 69.15%, 25.76%, and 1.68%, respectively–all of which likely began from an original strain that sequentially evolved and branched with chronic infection. The iterative nature of mutation acquisition has previously been demonstrated in the protease and reverse transcriptase domains of the *pol* gene of the HIV genome, as one of the driving forces behind resistance [19]. Indeed our sequencing data seems to suggest that acquired mutations follow a sequential path, though there may be situations where this is not the case.

Asymmetric PCR of the heterogeneous region was performed, yielding a 60 bp single-stranded target that was subsequently tested against five newly designed X-probes (**S6 and S7 Tables in** S1 File). To design the X-Probe, we incorporated the reverse complement of the consensus HIV hotspot sequence into the tunable “horizontal” region of the probe strand. Five protector strands were synthesized, incorporating progressively increasing numbers of mismatches, resulting in an interrogatable ΔG° range of approximately 18 kcal/mol. NUPACK was used to calculate the ΔΔG° for each probe-protector and probe-target sequence, allowing for approximation of hybridization yields for each target subpopulation.

**Fig 4:**
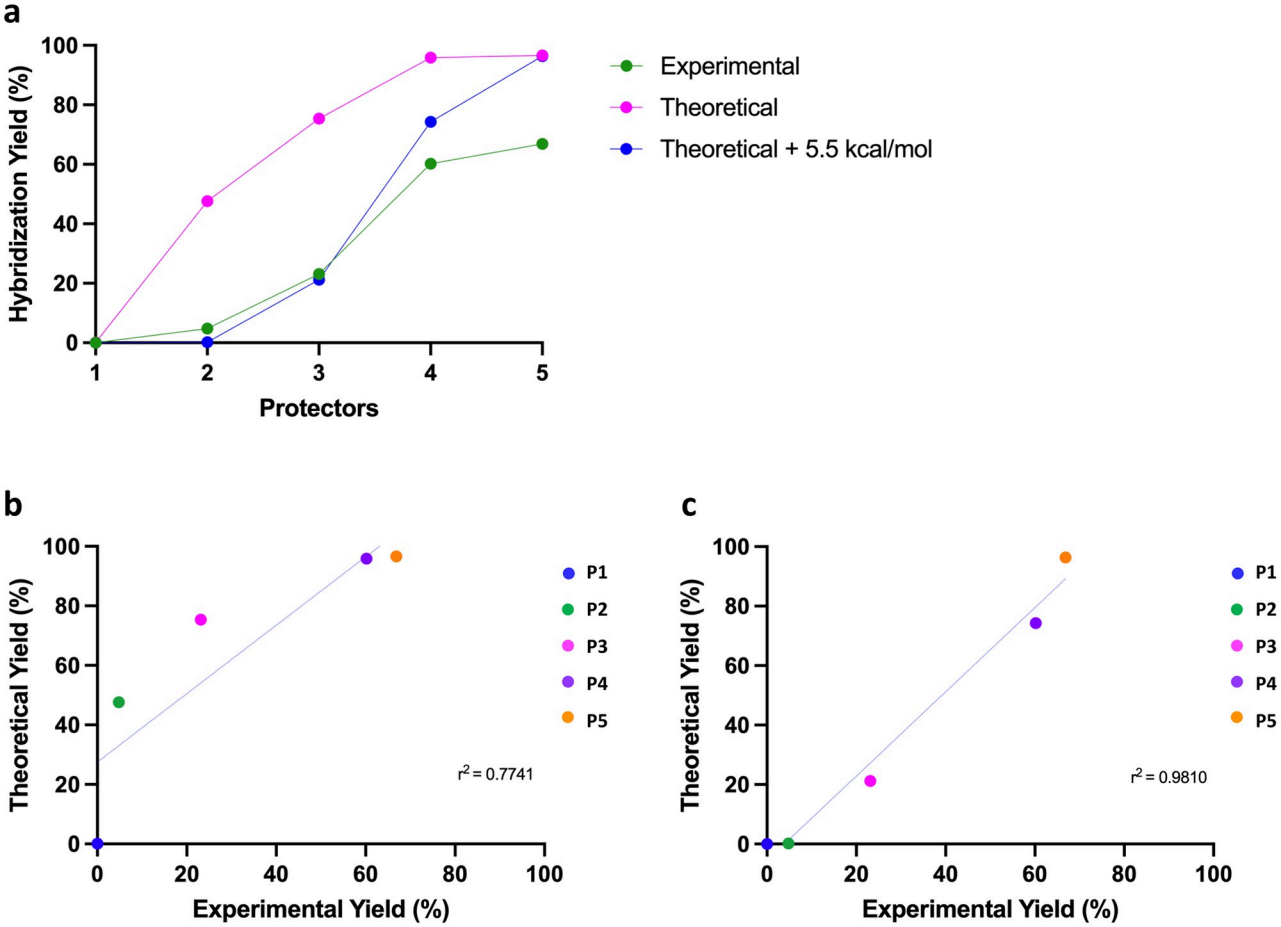
Evaluating performance of mismatch-tolerant protectors against hypermutable clinical correlates. Mismatch-tolerant X-Probes were designed and tested against an HIV-positive patient sample containing three HIV quasi-species with an iterative number of mutations in the *pol* gene. **A)** Experimental yields of displacement with five increasingly mismatch-tolerant X-probes are compared against theoretical yields, pre- and post- application of a 5.5 kcal/mol penalty. **B)** In the absence of the 5.5 kcal/mol penalty, the mismatch-tolerant protectors demonstrate moderate deviation from simulation (r^2^ = 0.77, Sy.x = 22.22). **C)** With the 5.5 kcal/mol penalty, experimental results correlate closely to theoretical estimates (r^2^ = 0.98, Sy.x = 7.060).

As shown in Fig 4a, the mismatch-tolerant protector design is capable of accurately resolving each unique HIV subtype as demonstrated by the incremental increase in signal corresponding to the relative distribution of each iteratively mutated sequence. The differential displacement observed with five increasingly mismatch-tolerant protectors initially demonstrates moderate deviation from simulation (r^2^ = 0.77, Sy.x = 22.22), as seen in Fig 4b. When a +5.5 kcal/mol penalty is imposed on the targets, however, the experimental results closely mirror simulation (r^2^ = 0.98, Sy.x = 7.060), as displayed in Fig 4c. The rationale behind this penalty is explored further in the Discussion section. This result, however, demonstrates the generalizability of the system even for complex solutions.

Without *a priori* knowledge of the sample’s composition, it is theoretically possible to back-calculate the number of unique and increasingly mutated sequences in a sample, as well as the approximate ΔG° of each probe-sequence complex, which in turn can be used to infer the number and possible identity of a variable number of SNPs. As noted above, marginal variations in ΔΔG° (defined as the ΔG° gap between [Probe-T] and [Probe-P]) significantly alter hybridization yields around null, producing sharp cutoffs in displacement within each ΔG° interval delineated by Eq 1. Successive mismatch-tolerant protectors can be designed to have narrow ΔG° gaps to afford better resolution (A-T mismatch), or large ΔG° gaps to span a large detection range (G-C mismatch). While this is an area of further investigation, one could theoretically infer the relative composition of the variably mismatched HIV *pol* targets in solution using S3 Fig, if the protectors were designed to yield sharper displacement cutoffs, i.e. 0% or 100%. For example, if P2 completely displaces in the presence of T1 and the prevalence of T1 in solution is 20%, we would expect a 20% rise in normalized signal. If P2 subsequently contributes no additional signal for T2 onwards, then P3 can be used to infer the relative contribution of T2 and so on and so forth.

## Discussion

We have demonstrated that a series of mismatch-tolerant protectors that span a sufficiently wide ΔG° range can be used to approximate the ΔG° of a probe-target complex, which in turn can be used to infer the identity of a target and its corresponding SNPs. We start by showing that X-probes represent a relatively cheap and efficient method to couple multiple mismatch-tolerant protectors with a single fluorophore-quencher strand complex obviating the need for repeated thermodynamic simulations of varying fluorophore-quencher paired sequences. The fluorophore- and quencher- functionalized oligonucleotides bind to “universal” portions of the probe and protector strands, which remain consistent across the various iterations of probes and protectors. For example, all protectors and probes used for the experiments described herein were functionalized to the same fluorophore- and quencher- labeled oligonucleotides, therefore requiring purchase of only one set of functionalized oligos for all experiments. In contrast, the standard probe-protector model–in which there are only two strands (a probe and protector) that are respectively coupled to a fluorophore or quencher–would require purchasing of separate labeled oligos for each variation of protector used. Hence, the use of X-probes is ideal for incorporating multiple tunable protectors at a lower cost, without affecting the predictability, hybridization efficacy, or kinetics of the system.

As shown in Fig 2, the hybridization of increasingly mismatched targets to toehold probes complexed to progressively mismatch-tolerant protectors closely follows prediction. Interestingly, experimental yields of displacement in Fig 2 never reach 100%, even when predicted by theory, instead demonstrating an asymptotic maximum of roughly 80% fluorescence relative to the positive control. This trend has been previously observed, however, when evaluating the kinetics of strand displacement in X-probes by synthetic targets via fluorometer analysis, and is postulated to be a result of misaligned hybridization, or non-specific interactions between the target and probe strands [17]. Further exploration of this effect may be warranted to fully elucidate the factors responsible for this reduced experimental maximum.

As demonstrated in Fig 3, the efficacy of our hybridization predictions are unaffected by the position of mismatches on either the protector or target strands. It should be noted that of all the representative targets and protectors tested, a few outliers were evident where experimental displacement diverged from prediction. This is especially clear in Fig 3b, where the M_16_ variation of the P_3_ protector yields unanticipated displacement against T_3_ target. Secondary structure analysis of the M_16_ variation of the P_3_ protector demonstrates that the mismatches unique to these strands encourage hairpin formation at the 3’ end of the protector near the target toehold (S2a Fig). As a result, the protector may have a propensity to self-dimerize as an inter-strand complex once strand displacement begins, incurring an additional thermodynamic penalty that permits the target to fully displace the protector. Interestingly, these protectors also demonstrate self-binding near the protector toehold region, perhaps hindering the ability of the protectors to re-bind the probe in equilibrium, leading to greater target-probe complexation stability (S2a Fig). In addition, the M_16_ protector strand also contains mismatches immediately adjacent to the multi-loop junction (S2b Fig), likely destabilizing the loop and allowing for more facile displacement of the protector from the probe. While these destabilizing mismatches are notably also present in protectors, M_13_, and M_18_, the combination of the effect of the mismatches with the above-described factors (that are specific to the M_16_ strand) may lead to the observed increase in protector displacement relative to theory seen with these cases. It should be noted that this effect is likely not immediately observed with M_16_ with T_1_, as the target is already thermodynamically favored to fully displace the M_16_ protector, and thus any added benefit to target-probe complexation does not significantly increase the expected yield. Other contributing factors that may lead to deviation from prediction include inaccuracies in NUPACK simulation, experimental error, and oligonucleotide synthesis errors, which may remain despite purification [20,21].

The results obtained in Fig 3a and 3d show yields of >100% for many of the reactions expected to show displacement (i.e. the mismatch protectors with T_1_ and the mismatch targets with P_4_). This yield is likely greater than the theoretical maximum (100%) due to occasional self-quenching of the positive control, which can occur as a result of the single labeled oligonucleotide aggregating or forming internal hybrid structures, both of which can result in contact-mediated quenching effects [22].

Altogether, the representative 101 combinations of mismatched protectors and targets follow predicted behavior closely demonstrating the robustness of the mismatch-tolerant protector system and its potential as a previously unfeasible, finely tuned mismatch tolerant hybridization system. The interrogatable range of ΔG°s can theoretically be extended using an increasing number of longer and sloppier protectors with various options on how to space these strands. While not within the scope of this manuscript, future areas of investigation include generation of a dose response curve of target to X-probe and the ability of the mismatch-tolerant protector system to predictably amplify target at minute concentrations using qPCR that would normally be undetectable using fluorophore-quencher. Though target was present in 2x excess of X-probe, we do not suspect varying concentrations of target will cause deviation from simulation given the above results.

Our system functions as expected even when multiple subpopulations containing variable numbers and identities of SNPs are present in a sample, as shown in Fig 4. Displacement of the mismatch-tolerant protectors closely adhered to prediction when a +5.5 kcal/mol penalty was imposed on the targets. Though the nature of the +5.5 kcal/mol penalty has not been thoroughly investigated, based on initial simulations, we believe this penalty is a product of the complex secondary structure formed by the 60 bp single-stranded HIV region of interest, which must be disrupted to displace the protector and bind the probe. Truncating the length of the target being assessed has the potential to overcome this issue, as the degree of self-binding outside of the region of interest is reduced with decreasing strand length.

While still proof-of-concept, the number of potential applications of the mismatch tolerant hybridization system is still unrealized. Perhaps most broadly, any hypermutable sequence or sequence with an uncharacterized set of SNPs can potentially be assayed using virtually any probe-based hybridization method in combination with our mismatch-tolerant protector system without the need to repeatedly test a sample using more costly approaches, such as sequencing. Further, unlike other hybridization-based assays—like microarrays—the system can tolerate mismatches in a finely tuned and precise manner without having to rely on other nonspecific mechanisms to modulate hybridization stringency, including lowering the hybridization temperature, adjusting the salt concentration, or inserting universal and/or more thermodynamically-favorable bases. Emerging technologies that utilize universal probes or primers also stand to benefit from such a design. As an example, the Universal Microbial Diagnostics system developed by previous members in our lab is a system that avails of the differential bindings of a set of universal, target-agnostic probes to various bacterial genomes as a means of identifying infectious bacterial species [10,23–27]. While the initial scheme utilized sloppy molecular beacon probes, implementation of the mismatch-tolerant toehold probes described herein may improve the specificity of probe binding, and therefore increase the overall efficacy of the identification platform. Though we do not directly compare the mismatch-tolerant probe system to sloppy molecular beacons herein, this may be an area for further investigation in the future.

## Materials and methods

### DNA oligonucleotides

The DNA oligonucleotides used in this study were purchased from Integrated DNA Technologies (IDT, Coralville, IA). All purchased oligonucleotides were purified via standard desalting, and pre-diluted in pH 8.0 IDTE buffer at a concentration of 100 μM. The fluorescent and quencher strands were ordered with a Carboxy X-Rhodamine (ROX) fluorophore and Iowa Black RQ quencher 3’ and 5’ modification, respectively.

### X-probe annealing protocol

X-probes were annealed at a ratio of 1:1.5:3:5 of fluorophore, probe, protector, and quencher respectively, using the following protocol: for a 100 μL reaction, added 10 μL 10 μM fluorescent strand, 15 μL 10 μM probe, 3 μL 100 μM protector, 5 μL 100 μM quencher strand, 50 μL 10x PBS, and 17 μL nuclease-free water. Strands were then run through the following thermocycling protocol on standard Eppendorf thermocyclers: incubation at 1) 95**°**C for 4 minutes, 2) 95**°**C for 1 minute, 3) ramp down by 1**°**C, 4) GOTO step 2 (repeat 75×), 5) 20**°**C for 1 minute.

### Fluorescent quantification studies

Fluorometer data from initial experiments testing mismatch-tolerant protector performance was gathered on the Horiba Scientific Fluoromax-4 Spectrofluorometer. The optimal excitation and emission wavelengths for the ROX fluorophore were set at 582 nm and 600 nm, respectively. Slit sizes were fixed at 4 nm for both excitation and emission, and integration time was set to 10s (per cuvette) for each 60s time point. Temperature was maintained at 37**°**C.

To achieve a final concentration of 10 nM of X-Probe, 12 μL of the 1 μM X-Probe solution was mixed with 1200 μL of 5x PBS in a standard quartz cuvette. Prior to spiking the X-Probe solutions with target, five minutes of fluorescent background (f_B_) data was attained. Cuvettes were then removed from the apparatus and 24 μL of 1 μM target solution (20 nM final concentration) was added after which each cuvette was capped, inverted 20 times to mix, and placed back in the machine to allow data acquisition to continue. For acquisition of positive control fluorescence, 12 μL of 1 μM of fluorophore was added to 1200 μL of 5x PBS in a standard quartz cuvette, and allowed to incubate for 5 minutes in the apparatus before being measured.

Fluorescence data from experiments testing the effect of global mismatch position on mismatch-tolerant protector performance were performed using a Bio-Rad CFX Connect Real-Time PCR Detection System (Bio-Rad, Hercules, CA). To set up the reaction, 10 μL of water, 10 μL of 1 μM target, 25 μL of 10x PBS + 0.2% Tween, and 5 μL of 1 μM X-Probe were combined and pipetted into a 96-well qPCR plate. Reactions were then allowed to incubate at 37°C for 1.5 hours. To process plates, end point ROX fluorescence was captured in the real-time PCR detection system using the following protocol: step 1) 37°C for one minute followed by fluorescent capture, step 2) GOTO step 1 × 5. RFU intensity readouts were then averaged over the five minutes. No background subtraction was performed.

### Preparation of HIV clinical samples

As an *in vitro* proof-of-concept, the efficacy of the mismatch-tolerant toehold-probe system was evaluated in the context of resolving quasi-species or viral subpopulations present in clinical HIV. Clinical HIV samples were collected from patients prior to receiving ART treatment (IRB #: 18-05-1929). For all patients, HIV RNA counts in plasma were high (>750,000 copies/mL). RNA was extracted from plasma samples using the Qiagen QIAamp Viral RNA Mini Kit (Qiagen, Germantown, MD). RNA to cDNA conversion was performed using the SMART cDNA Library Construction Kit by Clontech Laboratories, Inc (Takara Bio, San Jose, CA). Converted cDNA products were next column purified and subsequently amplified using universal primers designed relative to an internal consensus sequence derived from analysis of 800 patient samples, and corroborated against the HIV HXB2 reference sequence (GenBank: K03455.1). Phusion PCR amplification was performed via the following protocol: 10 uL of 5x Phusion HF buffer, 0.4 uL of 25 mM dNTPs, 0.5 uL of Phusion Hot Start polymerase, 0.4 uL of 100 uM primer mastermix, 10 uL of template, and 28.7 uL of water were combined to form a 50 uL reaction (Phusion PCR reagents were purchased from New England Biolabs, Ipswich, MA). The thermocycling protocol proceeded first with an incubation at 98°C for 30 seconds, followed by 30 cycles at 98°C for 10 seconds, 63°C for 30 seconds, and 72°C for 3 minutes, and a final elongation step at 72°C for 5 minutes. Samples were then column purified once more to remove PCR by-products and residual enzyme/buffer.

### Hotspot region amplification of cDNA

cDNA samples were again purified via column purification using a Zymo Research DNA Clean and Concentrator-25 kit (Zymo, Irvine, CA). Select 200–300 bp regions were then amplified using specific primers designed against various hotspot regions found in the patient-sample derived consensus sequence, and a high-fidelity polymerase (Phusion) purchased from New England Biolabs (NEB, Ipswich, MA). The primer pair sequences for the hotspot amplicon ultimately chosen for the clinical correlates experiment are provided here: 5’ – TAGAAGCAGAAGTTATTCCAGC – 3’ (forward) and 5’ – GATGAATACTGCCATTTGTACTG – 3’ (reverse). 10 μL PCR reactions were set-up using 2 μL 5x HF Buffer (NEB), 0.5 μL 10 mM dNTPs (NEB), 0.5 μL 10 μM forward primer, 0.5 μL 10 μM reverse primer, 0.2 μL Phusion polymerase, and 4.5 μL water. Reactions were pipetted directly into 0.2 mL PCR tubes, vortexed, and centrifuged down. The thermocycling protocol was performed on a Bio-Rad T100 Thermal Cycler as follows: step 1) 98**°**C for 3 minutes; step 2) 98**°**C for 0.5 minutes; step 3) 63**°**C for 0.5 minutes; step 4) 72**°**C for 1 minute; Step 5) Repeat steps 2–4 45 times; Step 6) 72**°**C for 5 minutes; Step 7) 4**°**C Hold. After performing PCR amplification, samples were again purified via column purification using the Zymo Research DNA Clean and Concentrator-25 kit. Two rounds of PCR amplification were performed in total (using the above protocol). Amplicons were analyzed via Polyacrylamide Gel Electrophoresis (PAGE), quantified using a Qubit dsDNA fluorometer (Thermofisher Scientific, Waltham, MA), and sent to Genewiz for Sanger sequencing (Genewiz, South Plainfield, NJ). Based on the Sanger sequencing results, four regions of the fourteen initially amplified (seven hotspot regions on two separate patient samples) were found to contain a sufficient distribution of mutations and were further processed for Next Generation Sequencing.

### Characterizing clinical isolates and designing and testing corresponding mismatch-tolerant probes

The four aforementioned PCR amplicons (analyzed via Sanger sequencing) were amplified once again using the same thermocycling protocol as used previously, and column purified. Post-amplification products were characterized via Bioanalyzer (Agilent, Santa Clara, CA) and quantified using the Qubit dsDNA Broad Range Assay kit. Samples were sequenced using the Next Generation Sequencing Amplicon-EZ service provided by Genewiz. A 30 bp segment in one of the four amplicons was found demonstrating significant SNP diversity (see **S5 Table in** S1 File; full sequencing results are available in S2 File). X-probes were subsequently designed against the consensus sequence; 5 protectors were designed incorporating increasing numbers of mismatches, and ΔG° values for all probe-protector and probe-target combinations were calculated in NUPACK (see **S6 Table in** S1 File). Iteratively mutated sequence breakdowns for the 30 bp segment were determined by evaluating all sequenced strands with a greater than 0.1% read rate over a 60 base pair region containing the 30 bp strand of interest. Asymmetric PCR was then performed on the sequenced sample to yield a 60-bp single-stranded “target” oligonucleotide (primers used are included in **S7 Table in** S1 File). 0.5 uL template DNA (corresponding to a final concentration of roughly 1 nM) was added to 1 uL 1 uM reverse primer and 2 uL 10 uM forward primer (a 1:20 ratio), 10 uL 5x Phusion HF Buffer, 1 uL 10 mM dNTPs, 1 uL Phusion Polymerase, and 34.5 uL water to form a 50 uL reaction. This sample was then placed in a Bio-T100 Thermal Cycler to undergo the following thermo-cyling protocol: 98°C for 3 minutes, followed by 70 cycles at 98°C for 30 seconds, then 63°C for 1 minute, and 72°C for 1 minute, and then lastly, one final step of elongation at 72°C. PCR products were cleaned using the Zymo Clean and Concentrator-DNA 25 kit, and characterized using the Qubit dsDNA Broad Range assay kit, Qubit ssDNA assay kit, and a Nanodrop. To quantify displacement of the X-probes by the target strand, a reaction mixture consisting of 1200 uL 5x PBS + 0.1% Tween was combined with 6 uL of 100 nM of each X-Probe (for X-Probes 1–5) and 12 uL single-stranded target (synthesized via asymmetric PCR). Reactions were allowed to incubate for 1 hour at room temperature before being analyzed for 15 minutes in the Horiba Scientific Fluoromax-4 Spectrofluorometer as described previously (Horiba Instruments Incorporated, Irvine, CA).

### Standard free energy calculation of yields

For all experiments, the general strand displacement reaction can be approximated as:

$$Probe\text{-}P_{x}+T_{y}⇋Probe+TP$$

Where P_x_ represents the complement strand or protector (P_1-5_), and T represents the target strand (T_1-5_). In this three-strand approximation, the region of the X-Probe immediately adjacent to the fluorophore/quencher label can be considered as contiguous with the probe and protector strands, and functions as the protector-toehold [17]. The region of the probe and protector strands complementary to the fluorophore and quencher strands, respectively, are not considered when determining hybridization efficacy. For the three-strand approximation, *K*_*eq*_ becomes

$$K_{eq}=\frac{{\left[TP\right]}_{eq}{\left[Probe\right]}_{eq}}{{\left[Probe-P\right]}_{eq}{\left[T\right]}_{eq}}$$

and can in turn be calculated from the difference between the free energies of the probe-complement complex and probe-target complex respectively (ΔΔG, where ΔΔG = ΔG_(Probe-T)_−ΔG_(Probe-P)_) such that

Keq=e−ΔΔGRT

where *R* represents the universal gas constant and *T* represents temperature. ΔG_(Probe-T)_ and ΔG_(Probe-P)_ were determined using NUPACK simulation software (version 3.0 for Figs 1–3, and version 4.0 for Fig 4) [28], but can generally be calculated based on sequence composition using the algorithm described in [16]. NUPACK input parameters vary based on experimental conditions; given our experimental setup, the temperature was set to 37°C, [Na^+^] was set to 0.685 M, [Mg^2+^] was set to 0.0 M, max complex size was set to 2 strands, and dangle treatment was set to “none.” Additionally, “AG” and “CT” base-pair combinations were used in place of the quencher (RQ) and fluorophore (ROX) to approximate the thermodynamic benefit of fluorophore-quencher binding.

In our system, if we let *x* = [*TP*]_*eq*_, we find that [*Probe* − *P*]_*eq*_ = [*Probe* − *P*]_0_ − *x*, [*Probe*]_*eq*_ = [*Probe*]_0_ + *x*, and [*T*]_*eq*_ = [*T*]_0_ − *x*. Given our initial conditions ([*Probe* − *P*]_0_ = 25 *nM*, [*Probe*]_0_ = 30 *nM* and [*T*]_0_ = 20 *nM*) we can rewrite *K*_*eq*_ as

$$K_{eq}=\frac{\left(x\right)\left(30+x\right)}{\left(15-x\right)\left(20-x\right)}$$

Solving for *x* allows for quantification of the hybridization yield *χ*, which can thusly be defined as the percent of probe-target relative to total probe concentration, or

$$\chi =\frac{x}{x+\left(15-x\right)}=\frac{x}{15}$$

## Supporting information

S1 Fig Kinetic traces of mismatch-tolerant X-probe strand—displacement reactions. Traces correspond to reactions of Targets T_1-5_ with Protectors (a) P_1_, (b) P_2_, (c) P_3_, (d) P_4_, and (e) P_5_. Kinetic traces demonstrate that mismatch-tolerant strand displacement reaction kinetics are in agreement with previously characterized toehold probe reaction rate constants. Further, the number of mismatches on the target or protector strand does not affect the rate of strand displacement, even when the mismatches are present on the target toehold, as is the case with T4 and T5.
(TIF)

S2 Fig Secondary structure analysis of the M_16_ variation of the P_3_ protector. **A)** Secondary structure simulations of the M_16_ protector in NUPACK reveal stable self-complexation near the 3’ end of the protector, adjacent to the target toehold region (shown in the green box), and near the 5’ protector toehold region (shown in the red box). These regions of self- binding show high equilibrium probability. **B)** Secondary structure simulations of the M_16_ X-probe structure in NUPACK reveal the potential destabilizing effect of the 3’ proximal mismatch in the multi-loop junction. This destabilization may facilitate opening of the X-Probe even in the presence of a less stable target (i.e. T_3_).
(TIF)

S3 Fig Predicted hybridization yields for X-probes against HIV subpopulations. Expected/theoretical yields for five protector sequences against the 3 HIV subpopulations. T_1_, T_2_, and T_3_, represent the most prevalent to least prevalent clinical subpopulations, respectively. Values are given in terms of percent yields.
(TIF)

S1 File Supplementary tables containing all probe, protector, and target sequences used in Figs 1–4, as well as the primer sequences used in Fig 4, are presented in the provided attachment. (DOCX)

S2 File Next-generation sequencing data for clinical HIV patient samples. The attached file contains the sequencing data for the HIV hotspot target used in Fig 4.
(XLSX)
