# Supplementary figures and images for: Simulation-guided tunable DNA probe design for mismatch tolerant hybridization
Source: PLoS One. 2024 Aug 22;19(8):e0305002. doi: 10.1371/journal.pone.0305002 (PMC11340886; doi:10.1371/journal.pone.0305002)

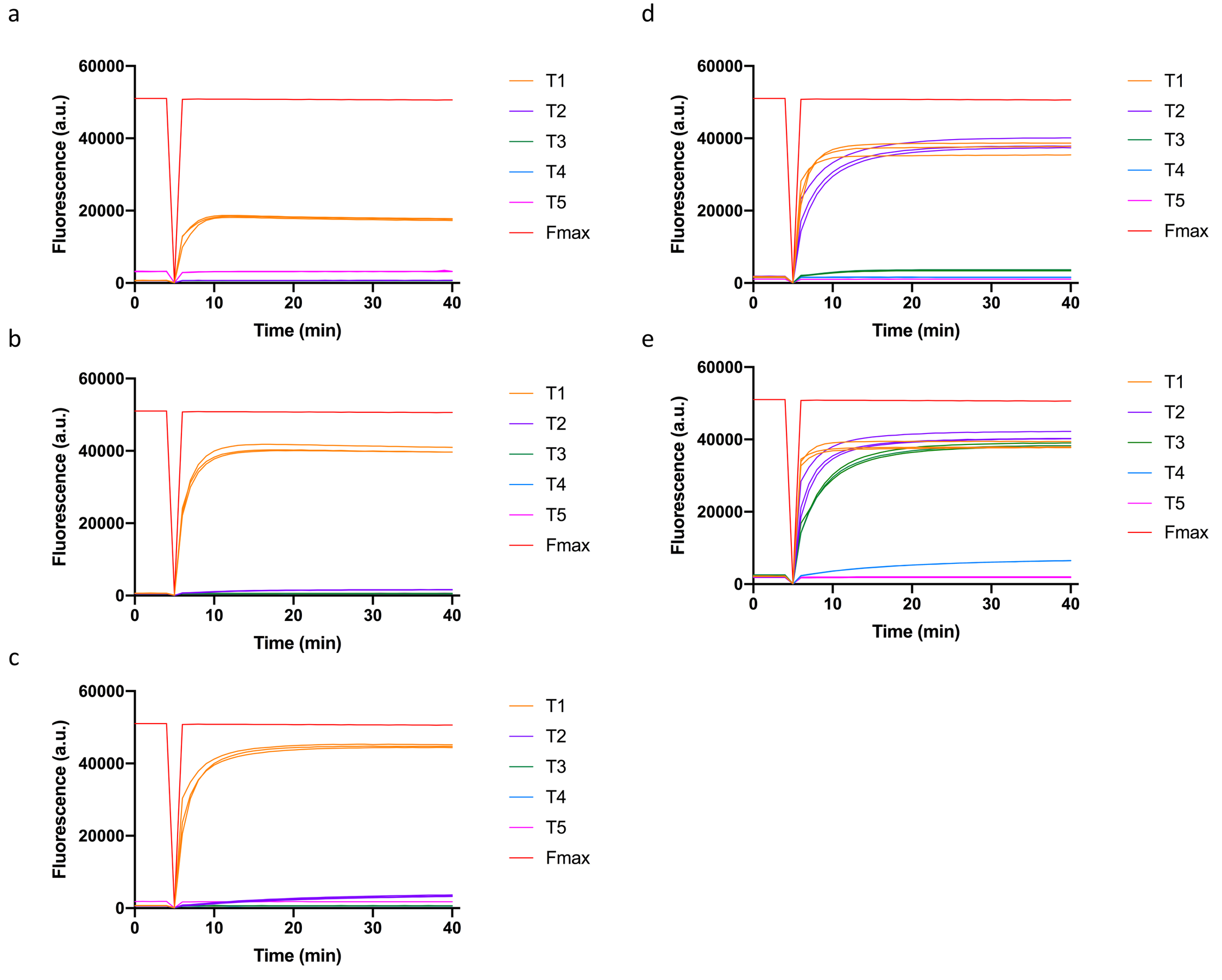

Supplement: S1 Fig — Traces correspond to reactions of Targets T1-5 with Protectors (a) P1, (b) P2, (c) P3, (d) P4, and (e) P5. Kinetic traces demonstrate that mismatch-tolerant strand displacement reaction kinetics are in agreement with previously characterized toehold probe reaction rate constants. Further, the number of mismatches on the target or protector strand does not affect the rate of strand displacement, even when the mismatches are present on the target toehold, as is the case with T4 and T5. (TIF) [file pone.0305002.s001.tif]

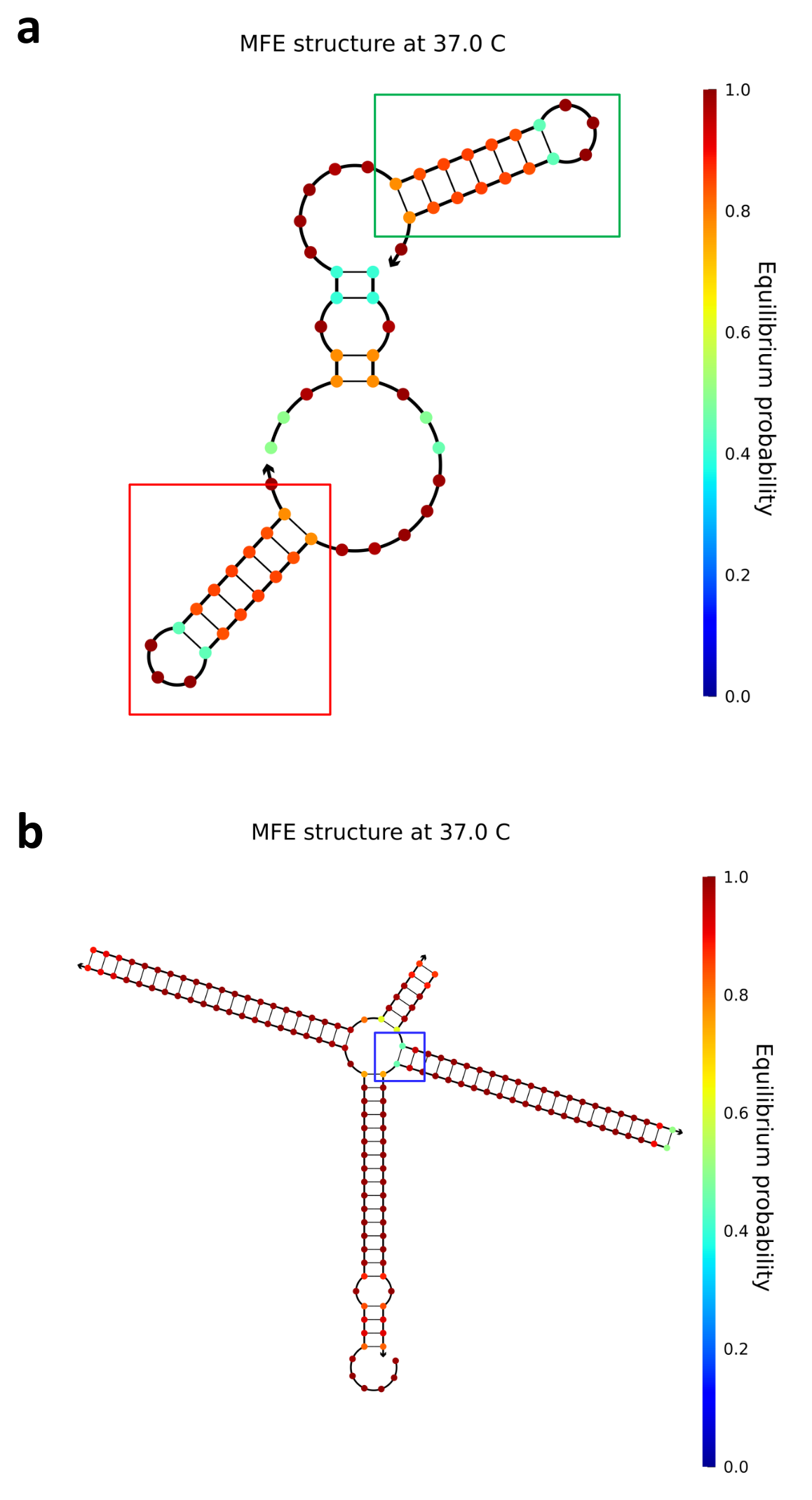

Supplement: S2 Fig — A) Secondary structure simulations of the M16 protector in NUPACK reveal stable self-complexation near the 3’ end of the protector, adjacent to the target toehold region (shown in the green box), and near the 5’ protector toehold region (shown in the red box). These regions of self- binding show high equilibrium probability. B) Secondary structure simulations of the M16 X-probe structure in NUPACK reveal the potential destabilizing effect of the 3’ proximal mismatch in the multi-loop junction. This destabilization may facilitate opening of the X-Probe even in the presence of a less stable target (i.e. T3). (TIF) [file pone.0305002.s002.tif]

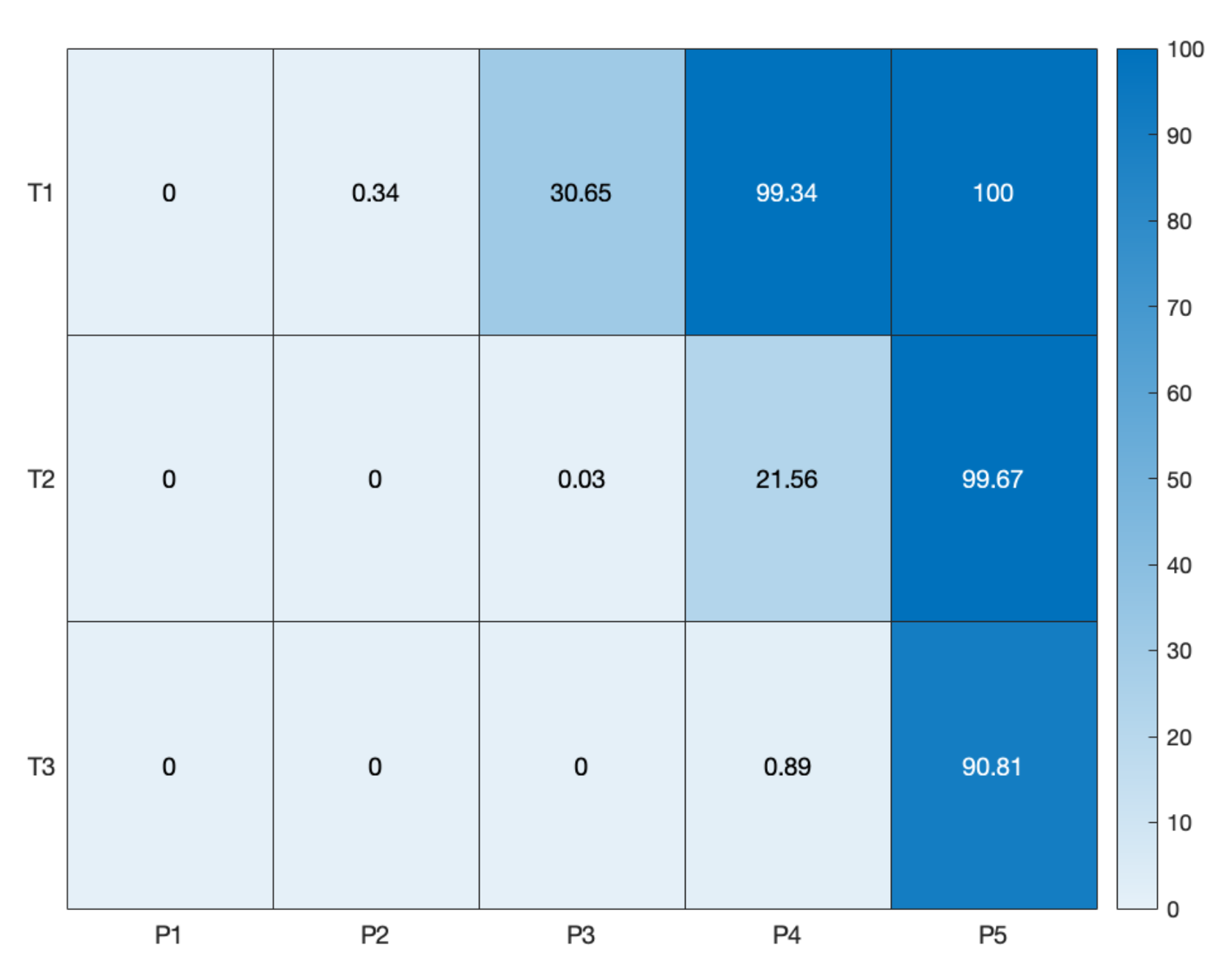

Supplement: S3 Fig — Expected/theoretical yields for five protector sequences against the 3 HIV subpopulations. T1, T2, and T3, represent the most prevalent to least prevalent clinical subpopulations, respectively. Values are given in terms of percent yields. (TIF) [file pone.0305002.s003.tif]
