# Supplementary material for: Simulation-guided tunable DNA probe design for mismatch tolerant hybridization
Source: PLoS One. 2024 Aug 22;19(8):e0305002. doi: 10.1371/journal.pone.0305002 (PMC11340886; doi:10.1371/journal.pone.0305002)
Supplement: S1 File — (DOCX) [file pone.0305002.s004.docx]

**Supplementary Tables:**

**Table S1a. Relevant X-Probe strands for Fig. 2 and 3.** The fluorophore (ROX) and quencher (RQ) are indicated in bold. Universal portions of the X-Probe are indicated in blue while the modifiable portion of the probe is indicated in green. Thermodynamically relevant portions of each strand – regions which are relevant for approximating hybridization efficacy – are underlined.

| **Strand** | **Sequence** |
| --- | --- |
| Probe | GCCCGCCCAAAATCTGTGATCTTGACTGGTCTACTATCCACGATTTAAC |
| Fluorescent Strand | GTTAAATCGTGGATAGTAGACTTCGCAC***ROX** |
| Quencher Strand | **RQ***GTGCGAACAGGTACATTTGCTCGTCCTT |

**Table S1b. ∆G of Probe+P_1-5_ and Probe+T_1-5_ for Fig.2.** Sequences show Protector strands (including the universal portions that are complementary to the quencher strand) and Target strands. Universal portions of the Protector strands are indicated in blue while the tunable portions are indicated in green. All Target strands are indicated in green as their entire sequence lengths are tunable. Thermodynamically relevant portions of each strand – regions which are relevant for approximating hybridization efficacy – are underlined. Mismatches in both the Protector and Target strands are indicated in red.

| **Sequence** | **dG (kcal/mol)** |
| --- | --- |
| P_1_: AAGGACGAGCAAATGTACCTGCAGTCAAGATCACAGATTTTGG | -37.8100 |
| P_2_: AAGGACGAGCAAATGTACCTGCAGTCAAGATCAC**T**GATTTTGG | -35.3700 |
| P_3_: AAGGACGAGCAAATGTACCTGCAGTC**G**AGATCAC**T**GATTTTGG | -33.0960 |
| P_4_: AAGGACGAGCAAATGTACCTGCAGTC**G**AGATCAC**T**GATTTT**A**G | -30.3320 |
| P_5_: AAGGACGAGCAAATGTACCTGCAGTC**G**AGATCAC**T**GAT**C**TT**A**G | -27.4606 |
| T_1_: ATGTCAAGATCACAGATTTTGGGCGGGCCA | -37.2900 |
| T_2_: ATGTCAAGATCACAGATTTTGGGC**T**GGCCA | -32.3500 |
| T_3_: ATGTCAAGATCACAGATT**C**TGGGC**T**GGCCA | -28.3000 |
| T_4_: ATGTCAAGATCACAGATT**C**TG**A**GC**T**GGCCA | -23.2600 |
| T_5_: ATGTCAAGATCACAGATT**C**TG**A**GC**T**GG**A**CA | -22.5800 |

**Table S2.** $\boldsymbol{\Delta\Delta}$**G of Probe+P_1-5_ and P+T_1-5_ for Fig. 2.** The $\Delta\Delta$Gs for all combinations of Probe + P_1-5_ against Probe + T_1-5_ are shown below.

| **Target** | **P_1_ (kcal/mol)** | **P_2_ (kcal/mol)** | **P_3_ (kcal/mol)** | **P_4_ (kcal/mol)** | **P_5_ (kcal/mol)** |
| --- | --- | --- | --- | --- | --- |
| **T_1_** | 0.52 | -1.92 | -4.19 | -6.96 | -9.83 |
| **T_2_** | 5.46 | 3.02 | 0.75 | -2.02 | -4.89 |
| **T_3_** | 9.51 | 7.07 | 4.80 | 2.03 | -0.84 |
| **T_4_** | 14.55 | 12.11 | 9.84 | 7.07 | 4.20 |
| **T_5_** | 15.23 | 12.79 | 10.52 | 7.75 | 4.88 |

**Table S3. Mismatch Protectors Used in Global Mismatch Position Experiments.** Sequences show Protector strands (including the universal portions that are complementary to the quencher strand). Universal portions of the Protector strands are indicated in blue while the tunable portions are indicated in green. Thermodynamically relevant portions of each Protector strand are underlined. Mismatches are shown in red. Note – Protectors M_4_, M_9_, M_13_, M_16_, and M_18_ incorporate mismatches in the universal portion immediately proximate to the loop to facilitate assessment of the effect of mismatches on loop stability.

| **Protector (Fig.3)** | **dG (kcal/mol)** |
| --- | --- |
| M_1_: AAGGACGAGCAAATGTACCTGCAGTCAAGATCAC**TC**ATTTTGG | -31.98 |
| M_2_: AAGGACGAGCAAATGTACCTGCAGTCA**GA**ATCACAGATTTTGG | -31.88 |
| M_3_: AAGGACGAGCAAATGTACCTGCAGTCAAGATCACAGATT**CA**GG | -33.37 |
| M_4_: AAGGACGAGCAAATGTACCTGC**TT**TCAAGATCACAGATTTTGG | -31.93 |
| M_5_: AAGGACGAGCAAATGTACCTGCAGTCAAGAT**GT**CAGATTTTGG | -32.11 |
| M_6_: AAGGACGAGCAAATGTACCTGCAGTCAAGATCACAG**CA**TTTG | -33.03 |
| M_7_: AAGGACGAGCAAATGTACCTGCAGTC**T**AGATCAC**G**GATTTTGG | -32.93 |
| M_8_: AAGGACGAGCAAATGTACCTGCAG**A**CAAGATC**T**CAGATTTTGG | -31.61 |
| M_9_: AAGGACGAGCAAATGTACCTGC**T**GTCAAGA**G**CACAGCTTTTGG | -30.00 |
| M_10_: AAGGACGAGCAAATGTACCTGCAGTCAAGAT**G**ACAGATT**G**TGG | -31.45 |
| M_11_: AAGGACGAGCAAATGTACCTGCAGTCAAGA**A**CACAGAT**A**TTGG | -31.70 |
| M_12_: AAGGACGAGCAAATGTACCTGCAGTCAAG**C**TCACAGA**G**TTTGG | -32.36 |
| M_13_: AAGGACGAGCAAATGTACCTGC**T**GTCAAGATCACAGATT**A**TGG | -32.20 |
| M_14_: AAGGACGAGCAAATGTACCTGCA**T**TCAAGATCACAGATTTT**C**G | -30.88 |
| M_15_: AAGGACGAGCAAATGTACCTGCAG**C**CAAGATCACAGATTTTG**A** | -32.95 |
| M_16_: AAGGACGAGCAAATGTACCTG**T**AGTCAAGATCACAGAT**C**TTGG | -30.53 |
| M_17_: AAGGACGAGCAAATGTACCTGCA**C**TCAAGATCACAGATTT**G**GG | -30.90 |
| M_18_: AAGGACGAGCAAATGTACCTGC**T**GTCAAGATCACAGATTTT**A**G | -32.60 |
| M_19_: AAGGACGAGCAAATGTACCTGCAG**G**CAAGATCACAGATTTT**T**G | -33.03 |

**Table S4. Mismatch Targets Used in Global Mismatch Position Experiments.** Sequences show the complete Target strands. Strands are in green to indicate that the whole sequence lengths of the Target strands are tunable. Mismatches are indicated in red.

| **Target (Fig.3)** | **dG (kcal/mol)** |
| --- | --- |
| N_1_: ATGTCA**TC**ATCACAGATTTTGGGCGGGCCA | -31.63 |
| N_2_: ATGTCAAGATC**TT**AGATTTTGGGCGGGCCA | -31.52 |
| N_3_: ATGTCAAGATCACAG**TC**TTTGGGCGGGCCA | -32.49 |
| N_4_: ATGTCAAGATCACAGATTT**CA**GGCGGGCCA | -31.19 |
| N_5_: ATGTCAAGATCACAGATTTTGG**CG**GGGCCA | -31.01 |
| N_6_: ATGTCAAGATCACAGATTTTGGGCGG**AG**CA | -34.63 |
| N_7_: AT**AC**CAAGATCACAGATTTTGGGCGGGCCA | -35.10 |
| N_8_: AT**T**TCAAGATCAC**C**GATTTTGGGCGGGCCA | -31.70 |
| N_9_: ATGT**T**AAGATCACAG**G**TTTTGGGCGGGCCA | -31.89 |
| N_10_: ATGTCA**T**GATCACAGATT**A**TGGGCGGGCCA | -31.26 |
| N_11_: ATGTCAAGA**G**CACAGATTTT**A**GGCGGGCCA | -30.50 |
| N_12_: ATGTCAAGATCACAG**G**TTTTGGGCGG**A**CCA | -32.15 |
| N_13_: ATGTCAAGATCACAGATT**A**TGGGCGGGCC**T** | -34.06 |
| N_14_: **T**TGTCAAGATCACAGATTTTGGGCGGG**A**CA | -36.38 |
| N_15_: A**C**GTCAAGATCACAGATTTTGGGCGG**C**CCA | -33.70 |
| N_16_: A**A**TTCAAGATCACAGATTTTGGGCGGG**A**CA | -34.59 |
| N_17_: ATG**C**CAAGATCACAGATTTTGGGCGGGC**T**A | -35.37 |
| N_18_: ATGT**T**AAGATCACAGATTTTGGGCGGGC**G**A | -33.88 |
| N_19_: ATGTC**T**AGATCACAGATTTTGGGCGGGC**T**A | -34.55 |

**Table S5. Percent Distribution of Unique HIV Viral Quasi-Species in a Human HIV Patient Sample.** Reads with a percent prevalence less than 0.1% were eliminated as they were below the reported Illumina sequencing error rate. Sequence similarities within the 60 bp region of interest were evaluated and re-tabulated to produce a condensed table of reads. New percent prevalence values were then calculated against said table.

| **% Prevalence** | **Sequence** |
| --- | --- |
| 69.15% | GTGGGCAGGGATTAAGCAGGAATTTGGCAT |
| 25.76% | GTGGGCAGGGATTAA**T**CAGGAATTTGGCAT |
| 1.68% | GTGGGCAGGG**G**TTAA**T**CAGGAATTTGGCAT |

**Table S6. X-probe strands for Elucidation of HIV Viral Quasi-Species in Human HIV Patient Sample.** The fluorophore (ROX) and quencher (RQ) are indicated in bold. Universal portions of the X-Probe are indicated in blue while the modifiable portions of the Probe and Protector strands are indicated in green. Thermodynamically relevant portions of each strand are underlined. Mismatches are indicated in red.

| **Strand** | **Sequence** |
| --- | --- |
| Probe | GCCAAATTCCTGCTTAATCCCTGCCCTGGTCTACTATCCACGATTTAAC |
| Fluorescent Strand | GTTAAATCGTGGATAGTAGACTTCGCAC***ROX** |
| Quencher Strand | **RQ***GTGCGAACAGGTACATTTGCTCGTCCTT |
| Protector 1 | AAGGACGAGCAAATGTACCTGCAGGGCAGGGATTAAGCAGGAA |
| Protector 2 | AAGGACGAGCAAATGTACCTGCAGGGCAG**A**GATTAAGCAGGAA |
| Protector 3 | AAGGACGAGCAAATGTACCTGCAGGGCAG**A**GATT**C**AGCAGGAA |
| Protector 4 | AAGGACGAGCAAATGTACCTGCAGGGCAG**A**GATT**C**AGCA**T**GAA |
| Protector 5 | AAGGACGAGCAAATGTACCTGCAG**T**GCAG**A**GATT**C**AGCA**T**GAA |

**Table S7. PCR primers for Asymmetric Amplification of HIV Hotspot Target.** The sequences for the target HIV hotspot amplicon used in Fig. 4, as well as the forward and reverse primers used in the asymmetric amplification of the amplicon, are provided below.

| **Primers** | **Amplicon** |
| --- | --- |
| F: CAGTTAAGGCCGCCTGTT | CAGTTAAGGCCGCCTGTTGGTGGGCAGGGATTAAGCAGGAATTT  GGCATTCCCTACAATC |
| R: GATTGTAGGGAATGCCAAA |  |

**Supplementary Attachment 1**. **Next-generation sequencing data for clinical HIV patient samples.** The attached file contains the sequencing data for the HIV hotspot target used in Fig. 4.
